# The Sweeping Train

**Published:** 1900-02

**Authors:** 


### THE SWEEPING TRAIN.

THE woman with sweeping train is here. If one takes the pains to consult those learned in the fashion of woman's dress, or if he cares to glance at the various magazines devoted to this important topic, he will learn that she is likely to remain some time. Now there are various reasons why a medical journal should interest itself in the question of woman's attire. If woman's dress is so unhygienically constructed as to be a menace to her health or the health of those about her, it becomes the duty of those better informed to acquaint her of the fact, and so thoroughly has this been done in regard to the corset that the woman who puts around her waist a tight and unyielding girdle does so with the full knowledge of the consequences. She harms no one but herself, and if she can stand it, the physicians who ultimately profit by her folly certainly ought to be able to.

Possibly, however, the woman with the train does not fully comprehend what she is doing when she permits herself to become an adjunct to the street cleaning department. And that she may no longer have the cloak of ignorance to cover her unwisdom the JOURNAL begs leave to inform her of the following facts: Dust collected in street cars, streets, and public places has been found to contain tubercle bacilli, pus organisms, tetanus bacilli, the bacillus of malignant edema, and the bacillus of diphtheria. These, madam, with others less harmful, your long skirt carries into your home, there to be shaken or brushed out and inhaled by more or less susceptible persons! Under favorable conditions the trailing dress may collect and carry indoors the virulent germs of almost any of the infectious diseases.

So much for the sanitary side of the question. There is another appeal which will carry more force. The omnipresent germ has come to be regarded as a bogey which the medical man uses to frighten grown-up children away from various kinds of foolishness. Cannot the lady with sweeping train be roused to a realisation of the eternal fitness of things? She is not out of place in her own or a friend's home or at various social functions where her dainty skirts trail over clean floors or carpets. Indeed, such dress worn on proper occasion adds grace and beauty to its wearer, but let her dress in harmony with her surroundings and spare her fellow creatures the distress of witnessing her desperate and usually vain attempts to hold her skirts clear of the filth of the street or the disgust of seeing a trailing skirt, the bottom of which is filled inches deep with dirt.

On its own authority the JOURNAL would not dare prescribe the proper length of a street skirt. The subject is frought with danger. The golf skirt, which in this latitude seems to be a modest garment, has been barred in the school-room by the school board of Louisville. A praiseworthy organisation, known as the Rainy Day Club, sends out from its headquarters in New York City, a circular which among other things describes the costume the club's members are required to wear on inclement days. "The skirt shall not be more than six nor less than four inches from the ground." The JOURNAL endorses this decree. It appeals to women to consider thoughtfully the subject of becoming street attire. Is not that woman best dressed whose garments are best adapted to the place and circumstances of the wearer?

